# NucEnvDB: A Database of Nuclear Envelope Proteins and Their Interactions

**DOI:** 10.3390/membranes13010062

**Published:** 2023-01-03

**Authors:** Fotis A. Baltoumas, Dimitrios Sofras, Avgi E. Apostolakou, Zoi I. Litou, Vassiliki A. Iconomidou

**Affiliations:** 1 Section of Cell Biology & Biophysics, Department of Biology, School of Sciences, National & Kapodistrian University of Athens, Panepistimiopolis, 15701 Athens, Greece; 2 Institute for Fundamental Biomedical Research, Biomedical Sciences Research Center “Alexander Fleming”, 34 Fleming St., 16672 Athens, Greece; 3 Laboratory of Molecular Cell Biology, KU Leuven, Kasteelpark Arenberg 31—Box 2438, 3001 Leuven, Belgium

**Keywords:** nuclear envelope, nuclear pore complex, membrane proteins, protein interactions, databases, biological networks, data visualization, functional enrichment

## Abstract

The nuclear envelope (NE) is a double-membrane system surrounding the nucleus of eukaryotic cells. A large number of proteins are localized in the NE, performing a wide variety of functions, from the bidirectional exchange of molecules between the cytoplasm and the nucleus to chromatin tethering, genome organization, regulation of signaling cascades, and many others. Despite its importance, several aspects of the NE, including its protein–protein interactions, remain understudied. In this work, we present NucEnvDB, a publicly available database of NE proteins and their interactions. Each database entry contains useful annotation including a description of its position in the NE, its interactions with other proteins, and cross-references to major biological repositories. In addition, the database provides users with a number of visualization and analysis tools, including the ability to construct and visualize protein–protein interaction networks and perform functional enrichment analysis for clusters of NE proteins and their interaction partners. The capabilities of NucEnvDB and its analysis tools are showcased by two informative case studies, exploring protein–protein interactions in Hutchinson–Gilford progeria and during SARS-CoV-2 infection at the level of the nuclear envelope.

## 1. Introduction

The nucleus of eukaryotic cells is one of the most important subcellular compartments, as it organizes, protects, and regulates the genome. It is surrounded by a distinct, double-membrane system called the nuclear envelope (NE), which separates the cytoplasm from the nucleoplasm and controls most of the macromolecules exchanged between them. The NE also contributes to the spatial organization of the nucleus [1]. The NE comprises the inner and the outer nuclear membranes (INM and ONM, respectively), the intermembrane space between them, the nuclear pore complexes (NPCs), and the nuclear lamina, a structural mesh—made of intermediate filaments—that covers the nucleoplasmic side of the INM. The area of the cytoplasm around the nuclear envelope is known as the perinuclear region [2]. The two membranes of the NE are not similar, either in their lipid composition or the proteins that associate with them. The ONM, which in mammalian cells is practically continuous with the endoplasmic reticulum (ER) membrane, shares a number of proteins with the latter [2]. On the other hand, the INM is vastly different, both in its structure and in the proteins it contains. Proteins that reside on it must specifically be targeted to it since the NPCs block the passive transport of membrane proteins from the ONM to the INM [3,4,5].

The most studied role of the NE is carried out by the NPCs, which are probably the largest protein complexes of the cell, and the sole passage for molecules to travel between the cytoplasm and the nucleus, either actively or via diffusion if they are small enough. The proteins that constitute the NPCs are transmembrane proteins that anchor the complex right between the INM and the ONM, and peripheral proteins that associate with the former and form the ring structure of the NPC and the functional barriers, that inhibit the ability of larger molecules to pass through the NPC indiscriminately [2]. Proteins located at other NE compartments, including the nuclear lamina and the two nuclear membranes, have also been found to participate in an enormous range of functions, from transcriptional regulation and chromatin binding to structural support of the nucleus. A characteristic example is given in the LINC (linker of nucleoskeleton and cytoskeleton) complex, where a trimeric SUN protein, which is anchored in the INM, binds with three KASH peptides or proteins which are anchored in the ONM. This interaction takes place inside the intermembrane space of the two membranes and is the main connection between the nucleoskeleton (which is bound by SUN proteins) and the cytoskeleton, which is bound to the KASH proteins [6]. However, most transmembrane proteins of the two nuclear membranes, collectively called nuclear envelope transmembrane proteins (NETs), have yet to be further investigated [7]. 

Apart from the above, a number of other proteins have also been found to localize to the NE. For example, a number of proteins that are normally found in the cell’s plasma membrane have been found to translocate in the NE; examples include receptor tyrosine kinases (RTKs) such as the epidermal growth factor receptor (EGFR) [8], G-protein-coupled receptors (GPCRs) such as adrenergic receptors [9,10], and various substrate transporters [11,12]. These proteins can be moved to the NE either as part of their canonical function or as a result of specific signaling stimuli, and regulate responses to various signaling pathways. Furthermore, their presence in the NE has been implicated in a number of diseases, including some types of cardiopathies [11,13,14], skeletal muscle weakness [15,16], and various neurodegenerative syndromes [17]. In mammalian cells, proteins of the ER may also be translocated to the NE’s outer membrane, as the two membrane systems are continuous [2]. Finally, a number of viral proteins target the host cell’s NE to facilitate the virus’s reproduction and biological functions or disrupt the cell’s canonical processes [18].

It is evident that, as in any biological entity, the proper function of the NE’s elements is strongly related to proteins’ interactions. In fact, it can be argued that the complexity of protein–protein interactions (PPIs) in the NE rivals that of the plasma membrane. However, despite their importance, the collective investigation of PPIs in the NE is limited, with most efforts focusing on established structures such as the NPCs and LINC complexes. In addition, there are no available up-to-date resources specializing in the proteins of the NE and their role. The only currently available database focusing on nucleus compartments, including the NE, is the Nuclear Protein Database (NPD) [19], an initiative to organize available data on novel nuclear proteins isolated using gene-trap and other technologies. However, the database focuses mainly on humans, mice, and rats, and has not been updated since 2009. Another, now-defunct example was the NR-RTK database [20], a protein–protein interaction network on the associations of RTKs with nuclear receptors in the human proteome; however, this resource has also been decommissioned. In contrast, the majority of NE-localized proteins and their interactions, including most NETs, remain understudied, with available annotation scattered in general-purpose resources such as UniProt [21], KEGG [22], Reactome [23], and the Human Protein Atlas [24].

To aid in the endeavor of exploring the proteomics of the NE, in this work, we present NucEnvDB, a publicly available database of NE proteins and their interactions. NucEnvDB contains a manually curated set of proteins found in the NE, each provided with annotation on their subcellular location, function, disease associations, and protein–protein interactions. In addition, the database contains viral proteins targeting the NE of their host cells, providing additional information on the impact viral infection may have on the nucleus’s structure. Finally, NucEnvDB offers an easy-to-use pipeline for the creation, visualization, analysis, and functional enrichment of protein–protein interaction (PPI) networks for NE proteins and their interacting partners. The capabilities of NucEnvDB are showcased through two indicative case studies, namely a disease-focused analysis of NE proteins participating in Hutchinson–Gilford progeria syndrome and a visualization of host–pathogen interactions involving NE-localized proteins of SARS-CoV-2. The NucEnvDB database is publicly available through http://thalis.biol.uoa.gr/nucenv-db/ (accessed on 27 December 2022).

## 2. Materials and Methods

### 2.1. Data Collection, Annotation, and Classification

To compile the dataset of NE proteins, the subcellular locations of the NE were initially identified, using the subcellular location scheme defined by the UniProt database [21]. In this system, each compartment is described using controlled vocabulary terms and assigned to a unique subcellular location identifier (SL-ID). A list with all terms related to the NE was compiled and is shown in Table 1. These terms were used to perform searches in UniProtKB/Swiss-Prot (release 2022_4) to isolate all manually annotated protein entries with known presence in the NE. For each entry, the protein name, gene name, sequence, subcellular location, and database cross-reference records were retrieved from their UniProtKB records. To provide additional functional annotation, the ontology terms assigned to these proteins were retrieved from Gene Ontology (GO) [25,26], using the proteins’ UniProtKB accession numbers (ACs) as search terms in the GOA database [27]. Protein–disease associations, where available, were retrieved from OMIM [28] and DisGeNET [29]. The dataset components were clustered based on their percentage sequence identity with the CD-HIT algorithm [30].

Protein–protein interactions for the proteins of the NE dataset were retrieved from the IntAct database [31] (Release 243, July 2022), using the proteins’ UniProtKB ACs as search terms. These included interactions between the NE proteins themselves, as well as interactions between NE proteins and other proteins (first neighbors). IntAct describes binary interactions between two components using multiple entries for each deposited experiment, each evaluated by a confidence score (Mi-score). For interactions represented by multiple entries, the entry with the highest Mi-score value was selected to represent the protein–protein complex [32]. To facilitate the creation of more robust PPI networks, the binary interactions between the first neighbors themselves are also retrieved from IntAct, using the same search criteria as the NE interactions.

### 2.2. Database Organization and Implementation

The collected data are organized in a relational MySQL database accessible through a web interface, built using HTML5, CSS3, and JavaScript. Server-side operations are mainly handled by PHP. The LiteMol PDB viewer [33] is used to enable the visualization of PDB [34] coordinates for entries with available 3D structures. Interfaces for BLAST [35] and HMMER3 [36] are provided, enabling sequence homology searches against the database’s components through pairwise alignments and profile hidden Markov models (pHMMs), respectively. To facilitate the creation, analysis, and visualization of PPI networks, an automated pipeline for performing network analysis on database contents was designed. The pipeline utilizes Cytoscape.js [37], a JavaScript library based on the popular Cytoscape network analysis suite [38] for network visualization, the NetworkX Python library [39] for performing topological analysis on the created networks, and the Markov clustering algorithm (MCL) for performing network clustering [40]. In addition, an interface for functional enrichment is implemented, utilizing WebGestaltR, an R package for connection to the WebGestalt enrichment tool [41].

### 2.3. Case Studies

To illustrate the capabilities of NucEnvDB and its analysis tools, two case studies were designed and executed. For the first case study, a review of the literature was conducted to find diseases related to the NE and its proteins. The example of Hutchinson–Gilford progeria syndrome was selected; the syndrome is connected to mutations of the *LMNA* gene, producing lamins A and C [42,43]. The NucEnvDB entry corresponding to *LMNA* and lamin A/C (AC: P02545) was used as input in the NucEnvDB network analysis tool to construct the network, involving all relevant interactions (NE–NE, NE–first neighbor, and first neighbor–first neighbor). A confidence score cut-off of 0.45 or higher was used to include only medium- or higher-confidence interactions [44], based on the distribution of confidence score values for the human NE proteins in NucEnvDB (Supplementary Figure S6A). The resulting PPI network was exported and analyzed with Cytoscape v.3.8 [38]. Topological analysis was performed using NucEnvDB’s network analysis pipeline, as well as NetworkAnalyzer [45] through Cytoscape. Finally, functional enrichment analysis of the network was performed using the NucEnvDB functional enrichment interface.

A second case study involved analyzing NE-localized, host–pathogen protein–protein interactions implicated in SARS-CoV-2 infection and COVID-19. The NucEnvDB entries corresponding to SARS-CoV-2 proteins localized in the host NE were used as input in the NucEnvDB network analysis tool to construct a PPI network, involving all relevant interactions (NE–NE, NE–first neighbor, and first neighbor–first neighbor). A confidence score cut-off of 0.30 was chosen to include as many SARS-CoV-2–human contacts as possible, as it was observed that the majority of COVID-19-related PPIs have relatively low IntAct Mi-scores (Supplementary Figure S6B). The network was filtered to include only SARS-CoV-2–human interactions and was imported for analysis to Cytoscape. Topological analysis was performed using NucEnvDB’s network analysis pipeline, as well as NetworkAnalyzer [45] through Cytoscape. Network clustering was performed through clusterMaker2 [46] using the GLay community detection algorithm [47] as it was found to perform better for this particular network compared to MCL. Functional enrichment analysis was performed using the NucEnvDB functional enrichment interface, using *Homo sapiens* as the reference background, the Benjamini–Hochberg (BH) false discovery rate (FDR) correction, and a significance threshold (*p*-value) of 0.05.

## 3. Results and Discussion

### 3.1. Database Components

**Figure 1:**
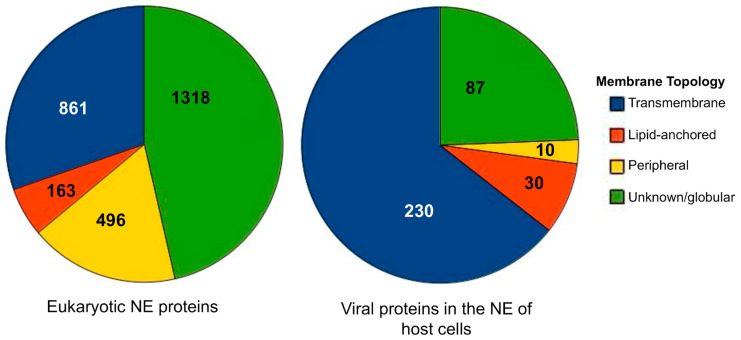
Distribution of NuEnvDB entries among membrane topology categories for nuclear envelope proteins (**left**) and viral proteins located at the NE of host cells (**right**). Each topology category corresponds to a different color (blue for transmembrane, red for lipid-anchored, yellow for peripheral, and green for unknown/globular proteins).

NucEnvDB currently contains 3195 entries, describing proteins with known presence in the nuclear envelope. Of these entries, 2838 correspond to eukaryotic NE proteins, coming from 168 species, while 357 correspond to viral proteins found at the NE of host cells, coming from 259 viruses. NucEnvDB entries are classified into four topology types with respect to their position in the membrane: transmembrane (spanning the lipid bilayer one or multiple times), peripheral (non-covalently bound to the membrane), lipid-anchored (covalently bound to a membrane lipid), or unknown/globular (has no distinct membrane topology). A distribution of the entries’ different topology types is presented in Figure 1, in the form of pie charts. As shown, in the case of eukaryotic NE proteins, the majority (*n* = 1318) of the entries are classified as unknown/globular and most likely interact with other parts of the NE using protein–protein interactions. The rest of the proteins bind directly to the membrane, with transmembrane NE proteins (*n* = 861) being the most abundant. In contrast to the above, viral NE proteins are primarily transmembrane (*n* = 230). Closer inspection of these viral proteins shows that they are primarily related to key processes such as hijacking the host cell’s replication/transcription/translation processes or virion nuclear egress, the first step of virion release from the infected cell.

A distribution of NucEnvDB entries among the different NE subcellular locations is given in Table 1 and Supplementary Figure S1. Table 1 shows the total number of entries per subcellular location, while Figure S1 shows the intersections among the different locations in the form of UpSet plots. As shown, there are two generalized subcellular locations, namely the “nuclear envelope”, for proteins directly bound to the NE, and the “perinuclear region”, for proteins located in the part of the cytoplasm immediately surrounding the envelope. These generalized location terms encompass all entries in NucEnvDB. In cases where the more detailed annotation is given on the specific position of a protein, its entry is also assigned to that particular position (e.g., a protein located in the nuclear membrane is assigned to that location, alongside the general term “nuclear envelope”). As shown by the distribution, a significant number of NE proteins (*n* = 1347) were assigned in this way, with the rest being assigned to the nuclear envelope itself (*n* = 1551). The latter contains 294 proteins with no further classification, indicating that their exact position in the envelope is not known; the rest (*n* = 1037) are classified as part of the nuclear membrane and its subcategories (ONM, INM, NPCs, and the lamina). A similar distribution is observed for the viral proteins as well, for which equivalent subcellular locations have been defined (e.g., “host nuclear envelope”, “host nuclear membrane”, etc.). However, as detailed information on their exact position is not available, the distribution of viral proteins in the NE appears to be more limited. Notably, an overlap (60 entries) exists between the perinuclear region and the NE components; these are primarily proteins found in other parts of the cell that are translocated to the NE. 

A total of 47,190 binary contacts featuring NucEnvDB entries are included, featuring either eukaryotic NE proteins or viral proteins targeting the NE. These involve 2040 interactions exclusively between NE proteins themselves, 40,825 interactions between NE proteins and other interactors, 4074 interactions between NE proteins and viral NE proteins, and 251 interactions between the viral NE proteins themselves. The confidence score (IntAct MI-score) of these interactions ranges from 0.15 to 0.99, with the average interaction score per organism being in the range of 0.35 to 0.50 (Supplementary Figure S2). Finally, the NucEnvDB entries are associated with a total of 5423 ontology terms (3746 biological processes, 913 molecular functions, and 764 cellular components), 794 phenotypes/diseases from OMIM, and 514 diseases from DisGeNET.

### 3.2. User Interface

NucEnvDB is publicly accessible through a user-friendly web interface, available at http://thalis.biol.uoa.gr/nucenv-db/ (accessed on 27 December 2022). Through the web interface, users can browse the database’s contents in four different manners: directly (“Browse Proteins”), by their location in the nuclear envelope (“Browse Envelope Locations”), by their organism (“Browse Organisms”), or by their association with Gene Ontology terms (“Browse Ontology Terms”). Database components can also be retrieved using search queries, both through a quick search form and through an “Advanced Search” page (Supplementary Figure S3). Searches can be performed using gene/protein names, UniProt ACs, organism names or NCBI taxonomy identifiers, assigned subcellular locations, and assigned protein topology. 

**Figure 2:**
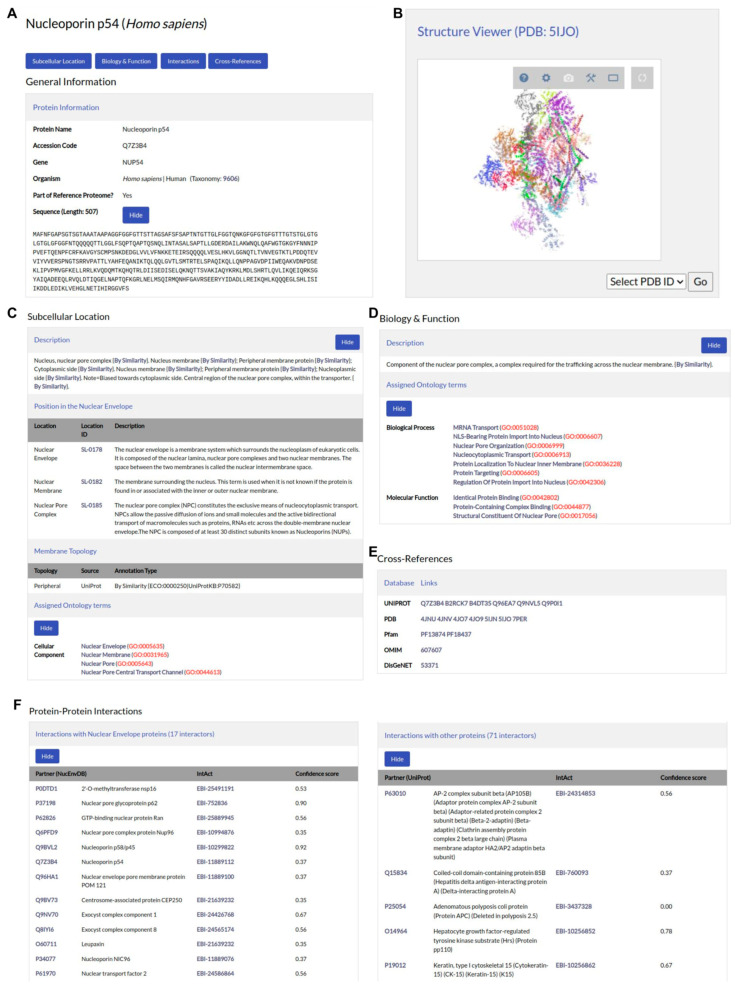
An example NucEnvDB entry. The human Nucleoporin p54 (AC: Q7Z3B4) is shown. (**A**) General protein information. (**B**) Structure viewer, showing the protein’s available 3D structures. Users may choose to load a structure using the dropdown menu at the bottom right corner of the viewer. (**C**) Subcellular location annotation. A detailed description of the protein’s subcellular location is given, accompanied by annotation of its membrane topology and the associated cell components by Gene Ontology. (**D**) Biological function annotation. (**E**) Cross-references to UniProt, PDB, Pfam, OMIM, and DisGeNET. (**F**) Protein–protein interactions, including the entry’s interactions with other NE proteins (**left**) and with proteins from other cell components (**right**). Each interaction is scored using a Confidence score, as assigned by IntAct (Mi-score).

An example entry is shown in Figure 2. In the database, each protein is represented using its primary UniProt AC as its NucEnvDB accession code. The entry page contains all relevant protein information, including a description of its subcellular location and its presence in the nuclear envelope, its function including all associated GO terms, its protein–protein interactions either with other NE proteins or with partners from other subcellular locations, and cross-references to major biological databases, including UniProt, PDB, Pfam, DisGeNET, and OMIM [21,28,29,34,48]. The structures of proteins with available 3D structures can also be visualized, using the LiteMol PDB viewer. Each individual entry, as well as the entire database, are available for download in TEXT, FASTA, and XML formats, through buttons at the top of the page. In addition, sequence clusters of the database’s entries at 40–90% sequence identity values are available for download in all the aforementioned file formats, through the database’s *Downloads* page.

In addition to the search options provided by the Quick and Advanced Search forms, users can also perform sequence homology searches using two alternative options: BLAST or HMMER. With BLAST, users can search one or more FASTA sequences of interest against the database’s sequence components through pairwise alignment, while with HMMER (Supplementary Figure S4), they can perform sequence searches against the database’s sequences (*phmmer*) or pHMMs of domains appearing in NucEnvDB’s entries (*hmmscan*) or search a multiple sequence alignment or pHMM of their own against the database’s sequences (*hmmsearch*).

**Figure 3:**
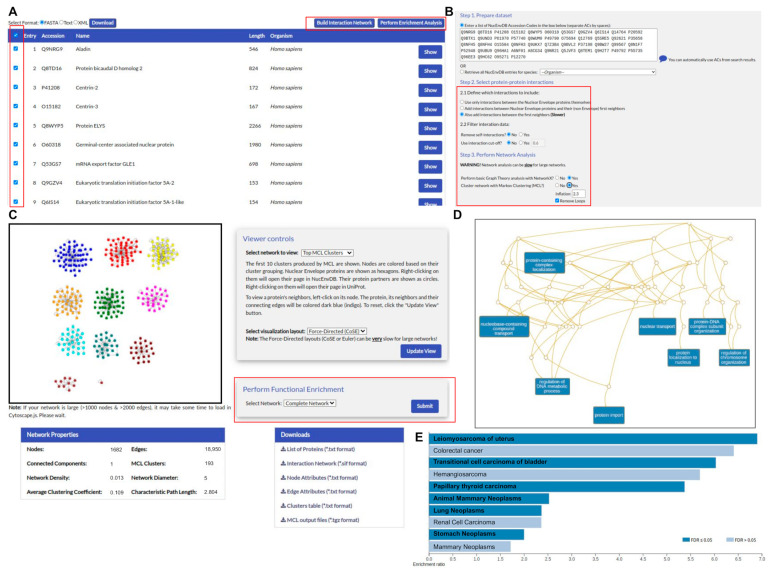
Example of PPI network analysis using the tools offered by NucEnvDB. (**A**) Through the Protein Browser, users may select one or more entries by clicking on the checkboxes to the left of the table. Selected entries can be downloaded using the buttons on the left, or sent for network analysis or functional enrichment using the buttons on the right. (**B**) Input form for the Network Analysis tool. Users can type/paste a list of NucEnvDB proteins (accession codes) into the input form, or select the NucEnvDB contents of an organism from the dropdown list. If the selection process described in (**A**) is followed, the input form is pre-loaded using the selected NucEnvDB entries. Users can select the type of interactions to include in the network from three options (only NE proteins, NE proteins, and first neighbors or full interactions, including the PPIs among first neighbors). They can also filter to exclude self-interactions or limit contacts to a specific confidence score (Mi-score) cutoff. Finally, they can choose to perform topological analysis or cluster their network with MCL. (**C**) The results panel, including a network viewer. Visualization can be performed for the entire network or for the top 10 MCL clusters, using a number of different layout algorithms. The topological properties of the network are given below the network viewer. The network and its components can be downloaded in formats compatible with Cytoscape from the Downloads panel, or sent for functional enrichment. (**D**) Functional enrichment with GO terms for the network components. Enriched GO categories are presented in the form of a directed acyclic graph. (**E**) Functional enrichment with disease associations for the network components. Results are given in a bar chart, with statistically significant terms colored deep blue.

NucEnvDB offers an automated, specially designed pipeline for the creation, analysis, and visualization of protein–protein interaction networks (Figure 3). Users can create networks featuring either the results of database searches or all the entries associated with an organism. The derived networks are subjected to topological analysis for the calculation of a number of metrics, including network density, characteristic path length, average clustering coefficient, node degrees, and the closeness and betweenness centralities. Clustering analysis can also be performed, using the MCL algorithm. Both the derived network and the top MCL clusters can be visualized through a network viewer powered by Cytoscape.js. Finally, the network analysis results can be used to perform functional enrichment analysis with WebGestaltR (Supplementary Figure S5), offering a choice of annotation options including Gene Ontology terms [26], metabolic pathways from KEGG [22] or Reactome [23], and, in the case of human PPI networks, disease associations from OMIM [28] or DisGeNET [29]. All network analysis components, including the PPI network itself, topological analysis metrics, MCL clusters, and enrichment analysis results are made available for download in file formats compatible with the desktop version of Cytoscape [38] for further analysis and visualization. The capabilities of the network analysis and functional enrichment tools are showcased in two case studies, presented in the following sections.

### 3.3. Case Study 1: Protein–Protein Interactions in Hutchinson–Gilford Progeria

Many diseases have been linked to NE proteins, with a characteristic example being Hutchinson–Gilford progeria syndrome. This condition is a rare disorder that leads to premature aging, starting within a year after birth [42]. The cause is typically a de novo mutation in the *LMNA* gene, causing the production of an abnormal protein often referred to as ”progerin” [43]. The *LMNA* gene produces mainly lamins A and C through alternative splicing—both primary components of the nuclear lamina, a network of intermediate filaments. A key moment in the maturation of lamin A (LMNA) is the farnesylation near its C-terminal that facilitates its association with the NE, followed by the cleavage by zinc metalloproteinase STE24 (ZMPSTE24) which releases the mature lamin A. In the case of the progeria mutation, lamin A retains the farnesylation site but lacks the cleavage site. This leads to the accumulation of progerin on the NE and is likely the cause of changes in nuclear architecture [49]. However, the disease phenotype was also found in mice in the absence of farnesylation [50], indicating that the missing segment affects the lamin’s function, potentially by disrupting its interactions with partner proteins.

**Figure 4:**
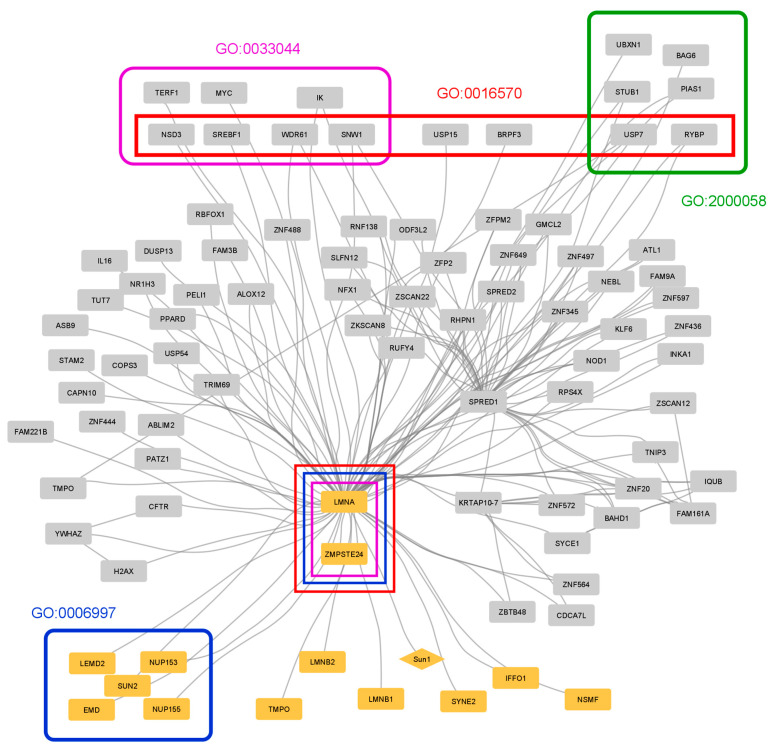
Interaction network for lamin A (LMNA), the protein responsible for Hutchinson–Gilford Progeria Syndrome. The interaction data and the GO annotation were collected using the Network Analysis and Functional Enrichment tools provided by NucEnvDB, respectively. This network is composed of 142 edges between 90 nodes, 14 NE proteins (orange), and 76 other proteins (gray). All are human proteins except for Sun1 (diamond), which is a *Mus musculus* protein, with its interaction indicated from experimental trials involving mice. Four GO terms regarding functions related to chromosomes (purple), the nucleus (blue), histones (red), and ubiquitin-dependent degradation (green) are shown with the associated proteins within frames of the same color. Most proteins only interact with LMNA; however, there are two other proteins with more than 10 interactions, KRTAP10-7 and SPRED1. (GO:2000058: regulation of ubiquitin-dependent protein catabolic process, GO:0016570: histone modification, GO:0033044: regulation of chromosome organization, GO:0006997: nucleus organization).

Progeria is only one of many diseases associated with lamin A [51], which is why this protein is of particular medical interest. To explore the interactomics of LMNA that are potentially related to Hutchinson–Gilford progeria, a PPI network focused on LMNA was created and analyzed (Figure 4, Supplementary Figure S7). A total of 89 proteins were found to interact with LMNA, 13 of which are NE proteins. Most proteins in the network interact only with LMNA, suggesting that the latter has the role of a connector between disparate proteins/pathways. Two proteins, in addition to LMNA, have a large number of interactions (degree > 10), SPRED1 and KRTAP10-7. SPRED1 is a membrane-associated protein member of the Sprouty/Spred family (regulators of the ERK/MAPK pathway) that was shown to participate in the regulation of the microtubule and actin cytoskeleton [52]. KRTAP10-7 is a keratin-associated protein; a recent study showed evidence that keratin filaments can have a supportive role in lamins and nuclear function [53].

Functional enrichment analysis revealed biological categories related to the function of chromosomes, histones, and the nucleus in general, as well as to ubiquitin-dependent degradation (Supplementary Table S1). An overlap between some of these categories was observed, as expected for such closely related processes. LMNA was involved in three of these along with another protein, ZMPSTE24. This is a protein very closely related to the function of LMNA as it is the metalloprotease responsible for the cleavage of LMNA. Additionally, enrichment analysis resulted in many diseases associated with these two proteins, including restrictive dermopathy (Supplementary Table S1). In fact, mutations in the ZMPSTE24 gene have been identified and associated with various diseases that share the premature aging characteristic of progeria [54]. Other NE proteins associated with the disease include EMD and atrial arrhythmia or NSMF, EMD, and LMNA linked to Emery–Dreifuss muscular dystrophy.

### 3.4. Case Study 2: Host–Pathogen Interactions at the Nuclear Envelope of Cells Infected with SARS-CoV-2

The severe acute respiratory syndrome coronavirus 2 (SARS-CoV-2, NCBI Tax. ID: 2697049) is the coronavirus strain responsible for the COVID-19 pandemic, a contagious respiratory disease responsible for more than 600 million confirmed infections and approximately 6.6 million deaths worldwide (November 2022 data) [55]. SARS-CoV-2 is a positive-sense, single-stranded RNA virus, with its genome having no nuclear phase and being replicated in the cytoplasm of host cells [56]. However, the virus has been indicated to also depend on the host cell’s nucleus, in order to facilitate proper replication [57]. A number of SARS-CoV-2′s non-structural proteins were found to alter nuclear import/export functions and impair the translocation of transcription factors involved in immune responses [58]. In addition, SARS-CoV-2 has been implicated with targeting host mRNAs, by inhibiting their release after transcription, blocking nuclear trafficking, and accelerating their degradation [59]. The above data indicate that at least some of the virus’s proteins target the NE of host cells, blocking PPI complexes implicated in the aforementioned canonical functions. Notably, the collection of viral NE proteins in NucEnvDB contains three proteins of SARS-CoV-2 that have been localized in the host NE area, namely replicase polyprotein 1a (R1A), also known as non-structural protein 11 (AC: P0DTC1), ORF7a (AC: P0DTC7), and 2’-O-methyltransferase nsp16, also known as replicase polyprotein 1ab (Rep) (AC: P0DTD1). All three proteins have been found in the host perinuclear region (SL-0382) and participate in interactions involving important NE elements, such as proteins of the NPCs and several NETs [60,61,62].

**Figure 5:**
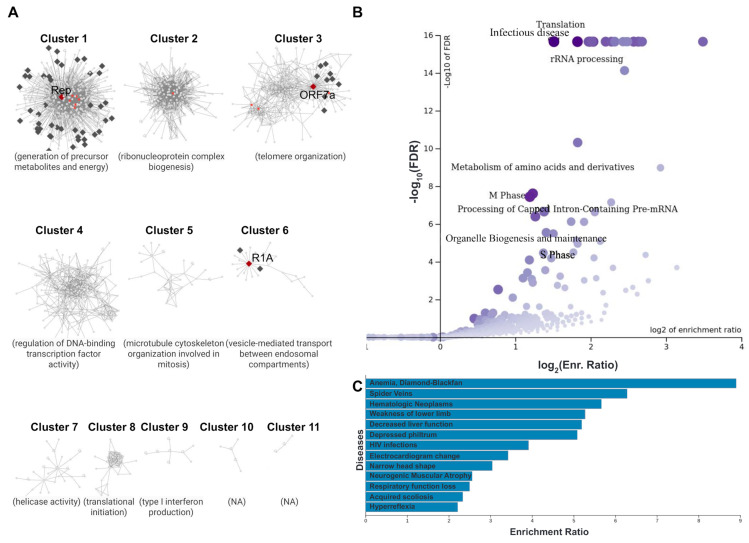
(**A**) The SARS-CoV-2–human interactome at the level of the nuclear envelope. The clustered network is shown. Proteins localized at locations of the NE (either human or viral) are shown as diamonds (♦), while proteins from other locations are shown as circles. Human proteins are colored dark gray for NE proteins and light gray for other interactors, while SARS-CoV-2 proteins are colored dark red for NE-localized proteins and bright red for other viral proteins. The labels of the three NE-localized viral proteins (R1A, ORF7a, and Rep) are shown. Each cluster is annotated with its top-ranking GO biological process term. (**B**) Functional enrichment with Reactome metabolic pathways. Enrichment results are shown in the form of a volcano plot. (**C**) Top disease associations produced from enrichment.

To further explore the processes involved in SARS-CoV-2 hijacking the host’s nucleus, a host–pathogen PPI network was created, featuring the aforementioned three SARS-CoV-2 NE-localized proteins and all their interacting partners (Figure 5, Supplementary Figure S8). A topological analysis of the network is given in Supplementary Table S2. Clustering of the network was performed with the GLay community detection algorithm (Figure 5A). Functional enrichment was performed for the entire network, as well as each separate cluster, using GO, DisGeNET, Reactome, and DrugBank to retrieve functional annotations. Enrichment results for the entire network are presented in Supplementary Table S3. The top-ranking GO term of each cluster is given in Figure 5A, while the top disease and metabolic pathway associations are presented in Figure 5B,C, respectively.

The network consists of 1052 nodes (proteins) and 4868 edges (interactions). A total of 13 of these nodes correspond to SARS-CoV-2 proteins, including the three NE-localized proteins (R1A, Rep, and ORF7a) and 10 other viral proteins. The rest of the nodes represent human interactors, including 55 NE proteins and 984 other human interactors. It is important to note that, contrary to standard PPI networks, this is a mixed graph containing intra-species interactions among the SARS-CoV-2 proteins themselves and the human proteins themselves, and host–pathogen interactions between the SARS-CoV-2 and human sets of proteins. This heterogeneity results in some discrepancies compared to analyzing a single-species PPI network, such as a high number of neighbors coupled with low density and low clustering coefficients. It is also probably the reason that MCL clustering, a standard in the analysis of most PPI networks, did not produce the desired results.

GLay community clustering of the network produced 11 groups with 3 or more members (Figure 5A). Four of these clusters (1, 2, 3, and 6) contained at least one SARS-CoV-2 protein, interacting with human proteins, while three (Clusters 1, 3, and 6) specifically contained viral proteins localized at the host NE (Rep, ORF7a, and R1A). Notably, these three clusters containing the host NE-localized SARS-CoV-2 proteins also contain the entirety of human NE proteins in the network, indicating that their implication in the interactome is directly connected to Rep, ORF7a, and R1A. The most prominent cluster (Cluster 1) is centered around the Rep protein of SARS-CoV-2 and also contains the majority of human NE proteins in the network, primarily nucleoporins and translocated substrate transporters. Its surrounding human proteins mainly participate in the generation of precursor metabolites and energy (GO:0006091). Similarly, Cluster 3 is centered around ORF7a and a few human NE proteins, primarily subunits of the integrator complex (INTS). The INTS is a large PPI complex that associates with the C-terminal domain of the RNA polymerase II large subunit and facilitates gene transcription. The cluster is connected, through functional enrichment analysis, to telomere organization (GO:0032200). Finally, Cluster 6 is a small group, centered around R1A in a complex with TMEM33 and unconventional myosin VI (MYO6). Its main biological process seems to be the vesicle-mediated transport between endosomal components (GO:0098927). In addition to the above, another prominent group is Cluster 4. Although it does not contain any SARS-CoV-2 proteins, it was found to participate in the regulation of DNA-binding transcription factor activity (GO:0051090), a process directly related to gene expression. 

In addition to the per-cluster analysis referenced above, the entire PPI network was subjected to functional enrichment analysis and annotation. The metabolic pathway, disease, and chemical compound terms are given in Supplementary Table S2 and Figure 5B,C. The top enriched metabolic terms are given in the form of a volcano plot in Figure 5B, and primarily involve processes commonly associated with viral infection and multiplication, such as RNA processing, cell cycle phases and metabolite/amino acid processing. The top associated disease terms are given in Figure 5C. Notably, these include pathological conditions that have been directly related to COVID-19, such as respiratory function loss [62], decreased liver function [63], and lower limb weakness [64]. In addition, the analysis highlighted diseases that have been found to result in complications when coupled with COVID-19, such as Diamond–Blackfan anemia [65] and neurogenic muscular atrophy [66]. Finally, enrichment analysis with chemical compounds from DrugBank highlighted artenimol (ID: DB11638), phenethyl isothiocyanate (ID: DB12695), and NADH (ID: DB00157). While the latter is a standard coenzyme in eukaryotic metabolism, the former two are drugs with potential applications to COVID-19 treatment. The active substance of artenimol is a drug originally designed to treat malaria. Its active substance is artemisinin, which has been proposed to combat SARS-CoV-2 infection by inhibiting its invasion, and replication [67]. Phenethyl isothiocyanate, an anti-cancer drug, has also been proposed as a potential candidate, through multi-omics bioinformatics analyses for the repurposing of drugs [68]. 

Overall, clustering and enrichment results are in agreement with what is known about SARS-CoV-2 influencing the host’s processes, i.e., targeting and disrupting gene transcription host mRNA facilitation and transcription factor activity (Clusters 3 and 4) [59], disruptions in metabolite production (Cluster 1) [69], and hijacking the cell’s vesicle transport system (Cluster 6). What is more, they provide a potential molecular basis for these effects, as they seem to be primarily focused on the NE-localized proteins of the virus. Whole-network enrichment also highlights pathological conditions that have been implicated in COVID-19 severity, as already stated. These observations essentially highlight the importance of host–pathogen interactions at the nuclear envelope as a key element in SARS-CoV-2 infection and COVID-19 progression.

## 4. Conclusions

We have presented NucEnvDB, a publicly available database of NE proteins and their interactions. The existence of such a manually annotated dataset can be utilized in large-scale analyses of the NE and its proteomics, including the exploration of host–virus interactions. In addition, the network analysis and functional enrichment tools offered by the database can be especially useful in easily constructing, analyzing, and annotating PPI networks for NE components of biomedical interest. The presented case studies demonstrate the capabilities of NucEnvDB in producing ready-to-use analysis results, both for biological networks and for functional annotation.

To our knowledge, NucEnvDB is currently the only available resource focused on the nuclear envelope and its protein components. To keep up to date, the database will be annually updated with new data, including new NE-localized proteins both from eukaryotic organisms and from viruses, as well as their associated interactions. Furthermore, future updates to the NucEnvDB website will include implementations of additional tools for the analysis and visualization of NE proteins, their interactions, and their functional role. Given the rising interest in studying the nuclear envelope, a previously unattended subcellular component, we expect NucEnvDB to be a valuable resource for genome-wide and/or proteome-wide analyses and, potentially, the design of novel prediction algorithms aimed at identifying nuclear envelope proteins.

## Figures and Tables

**Table 1:** List of subcellular locations assigned to the Nuclear Envelope. The name, UniProt identifier (SL-ID), and description of each location are given, as well as the number of NucEnvDB entries that have been assigned to each term.

Subcellular Location	SL-ID	Description	Proteins
Nuclear Envelope	SL-0178	The complex membrane system that surrounds the nucleoplasm. It is composed of two membranes, the nuclear lamina, and nuclear pore complexes. The space between the two membranes is called the nuclear intermembrane space.	1551
Nuclear Membrane	SL-0182	The membrane surrounding the nucleus. This term is used when it is not known if the protein is found in or associated with the inner or outer nuclear membrane.	1037
Nuclear Inner Membrane	SL-0179	The inner membrane of the nucleus is the membrane which separates the nuclear matrix from the intermembrane space.	211
Nuclear Outer Membrane	SL-0183	The outer membrane of the nucleus is the membrane facing the cytoplasm.	89
Nuclear Intermembrane Space	SL-0184	The nuclear intermembrane space is the space between the inner and outer nuclear membranes.	4
Nuclear Pore Complex	SL-0185	The nuclear pore complex (NPC) constitutes the exclusive means of nucleocytoplasmic transport. It is composed of at least 30 distinct subunits known as nucleoporins (NUPs).	389
Nuclear Lamina	SL-0180	The nuclear lamina is a meshwork of intermediate filament proteins called lamins and lamin-binding proteins that are embedded in the inner nuclear membrane.	32
Perinuclear Region	SL-0198	The perinuclear region is the cytoplasmic region just around the nucleus.	1347
Host Nuclear Envelope ^1^	SL-0415	Equivalent of SL-0178 for viral proteins that target the nuclear envelope of host cells.	93
Host Nuclear Membrane ^1^	SL-0182	Equivalent of SL-0178 for viral proteins that target the nuclear membrane of host cells.	85
Host Nuclear Inner Membrane ^1^	SL-0419	Equivalent of SL-0179 for viral proteins that target the inner nuclear membrane of host cells.	57
Host Nuclear Outer Membrane ^1^	SL-0446	Equivalent of SL-0183 for viral proteins that target the outer nuclear membrane of host cells.	0
Host Nuclear Lamina ^1^	SL-0416	Equivalent of SL-0180 for viral proteins that target the nuclear lamina of host cells.	7
Host Perinuclear Region ^1^	SL-0382	Equivalent of SL-0198 for viral proteins that target the perinuclear region of host cells.	231

^1^ These terms are defined for viral proteins that appear in the nuclear envelope-related subcellular locations of infected host cells.

## Data Availability

NucEnvDB is available through http://thalis.biol.uoa.gr/nucenv-db/ (accessed on 27 December 2022). All data presented in this work can be downloaded through the database’s *Downloads* page.

## Supplementary Materials

The following supporting information can be downloaded at: https://www.mdpi.com/article/10.3390/membranes13010062/s1, Supplementary File S1: Supplementary Figures and Tables.
