# Supplementary material for: NucEnvDB: A Database of Nuclear Envelope Proteins and Their Interactions
Source: Membranes (Basel). 2023 Jan 3;13(1):62. doi: 10.3390/membranes13010062 (PMC9861991; doi:10.3390/membranes13010062)
Supplement: Supplementary file 1 [file membranes-13-00062-s001.zip › membranes-2121135-supplementary.pdf]

NucEnvDB: a database of nuclear envelope proteins and their interactions

Supplementary Material

Supplementary Figures

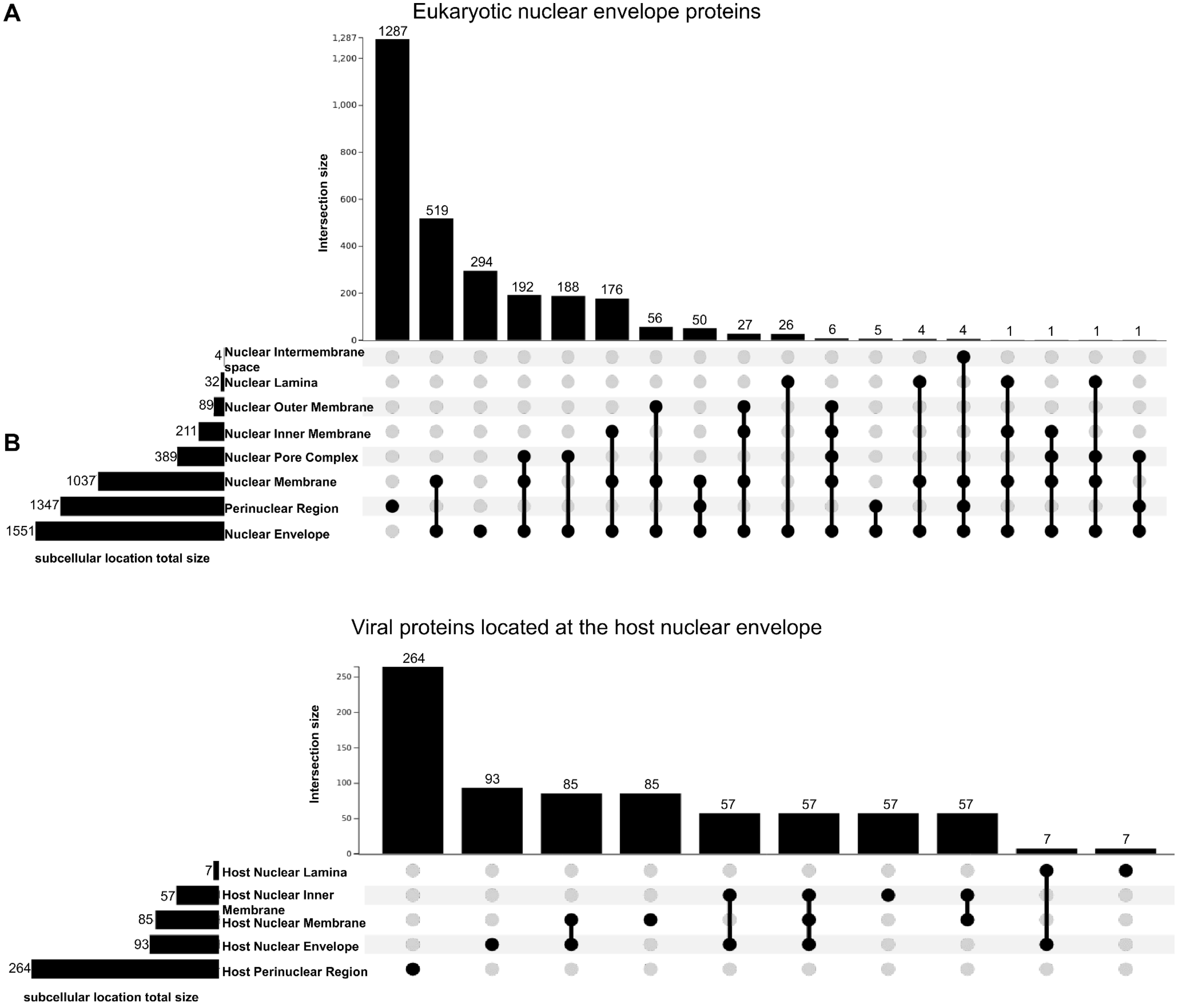

Figure S1. Distribution plots of NuEnvDB entries among subcellular locations for nuclear envelope proteins (A) and viral proteins located at the NE of host cells (B). The distributions are presented in the form of UpSet plots. The total size of each category is represented through the horizontal bar chart at the left of the panel. The various intersections among different categories are represented by the chart at the bottom of the panel, with each category shown as a dot and intersecting categories connected by straight lines. The sizes of the intersection sets are represented by the vertical bar chart at the top of the panel.

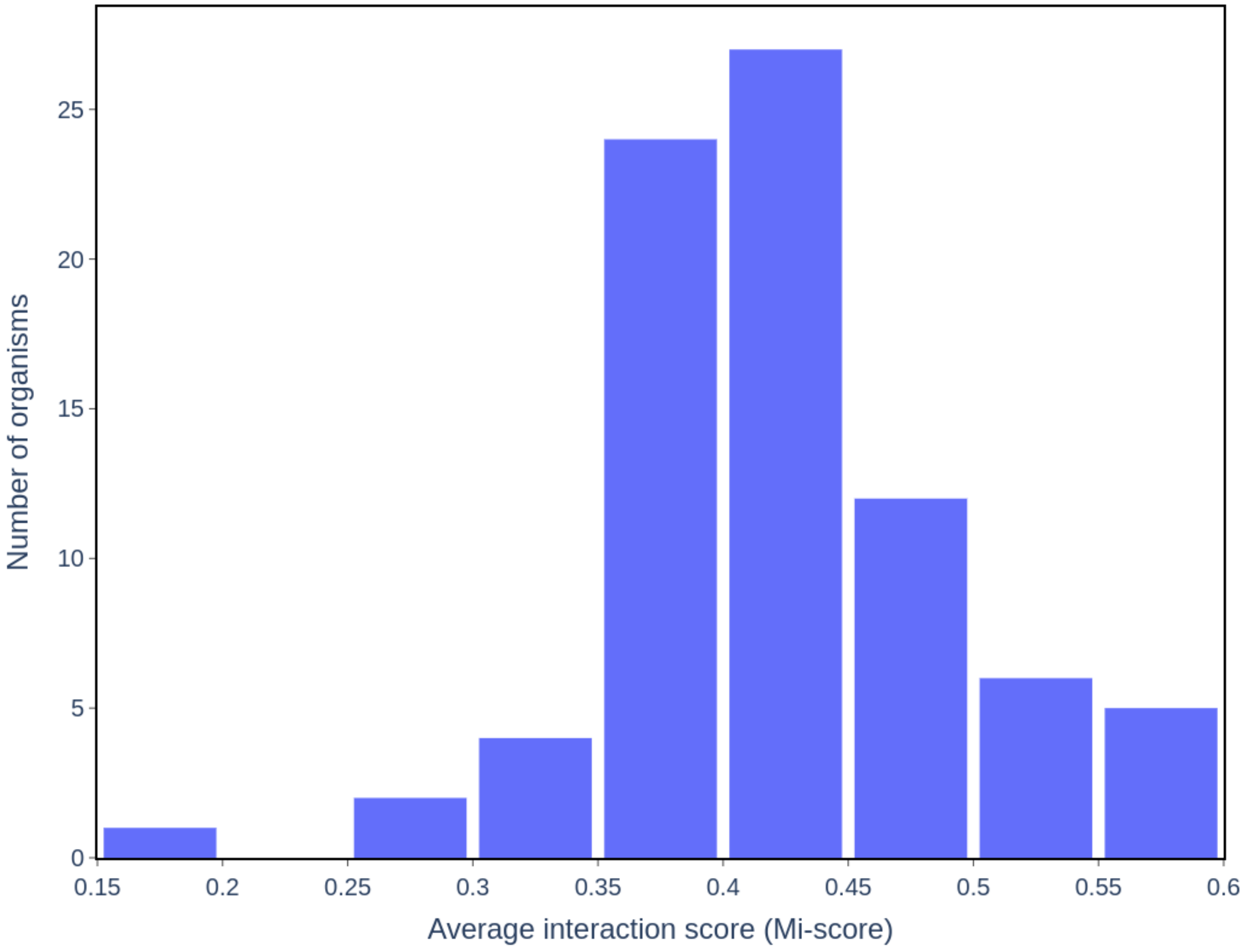

Figure S2. Histogram plot showing the distribution of the average interaction confidence score (Mi-score) per organism in NucEnvDB. The horizontal axis represents the mean Mi-score per organism (NCBI Taxonomy ID), grouped into intervals of 0.05, while the vertical axis shows the number of NucEnvDB organisms having an average Mi-score in that area.

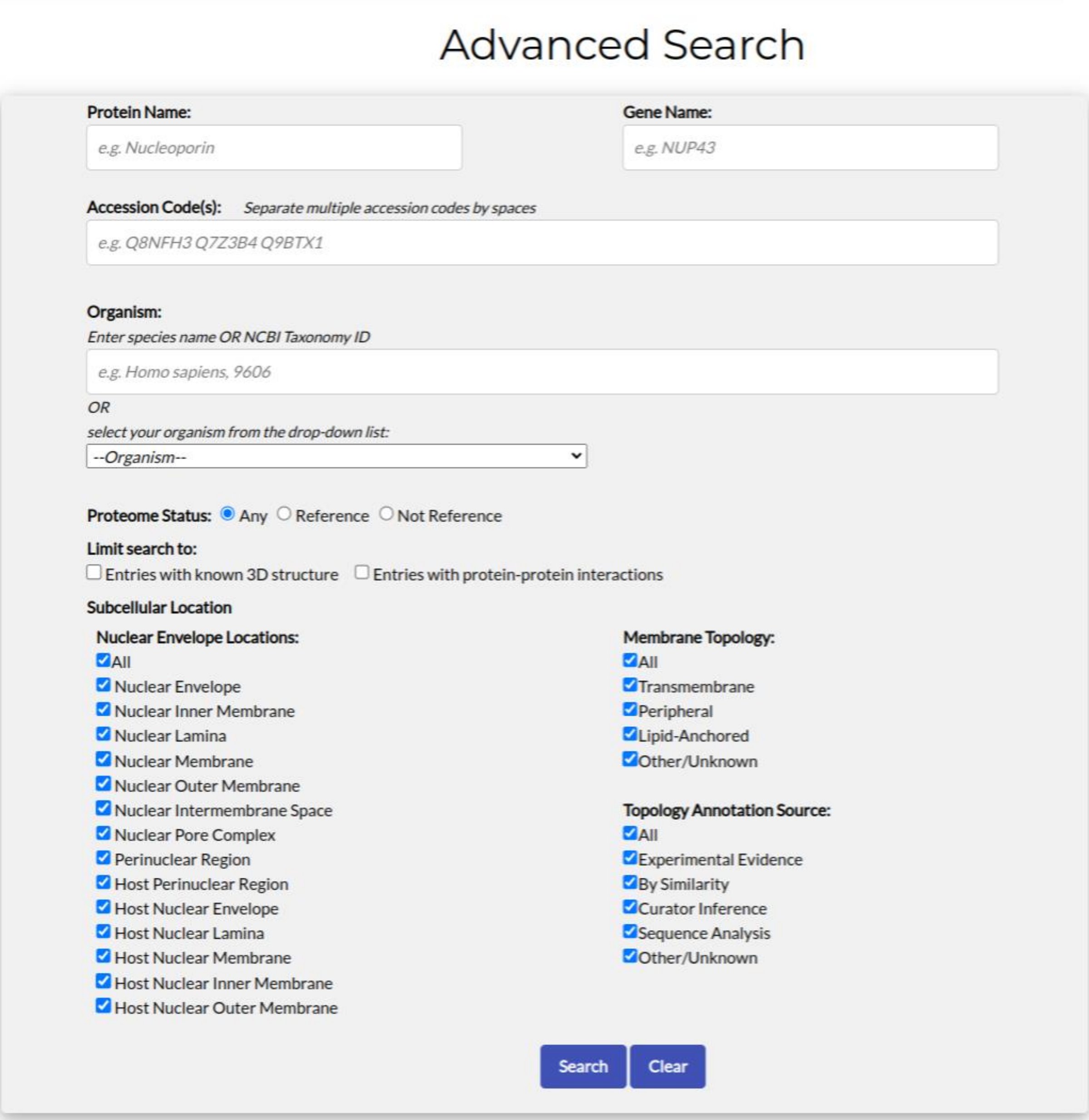

Figure S3. The Advanced Search input form. Users can perform searches with protein or gene names, by submitting one or more UniProt ACs, or by searching a specific organism. They can also limit their search to entries with 3D structures or protein-protein interactions, or with proteins having a specific membrane topology or belonging to a specific part of the envelope.

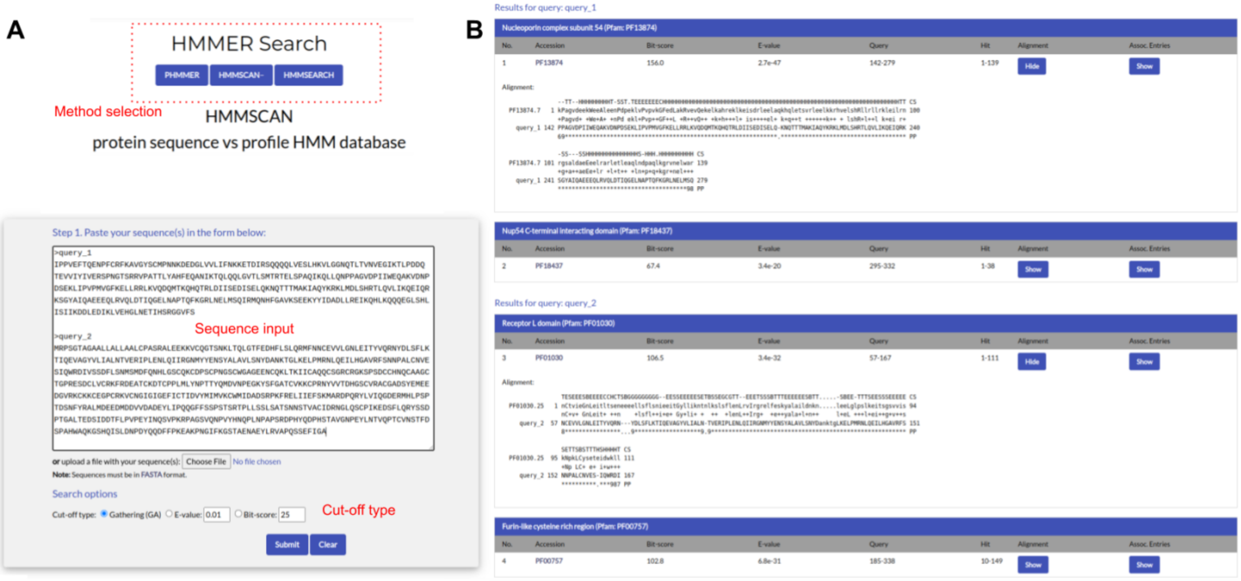

Figure S4. A) The HMMER search input form. At the top of the page, users can select the search method among three options: Sequence vs Sequence (PHMMER), Sequence vs pHMM (HMMSCAN) or pHMM/alignment vs Sequence (HMMSEARCH). For each option, a sequence input window is provided, in which queries can be uploaded in the FASTA file format. In addition, settings to define the search cut-offs are provided, to help refine the search according to the user's needs. B) Example results with HMMSCAN. For each hit, the alignment between the query and the hit is provided in the HMMER alignment format. Alignment properties, including the bit-score, E-value and sequence ranges of the query and hit are also provided. The results can be downloaded in the HMMER file format.

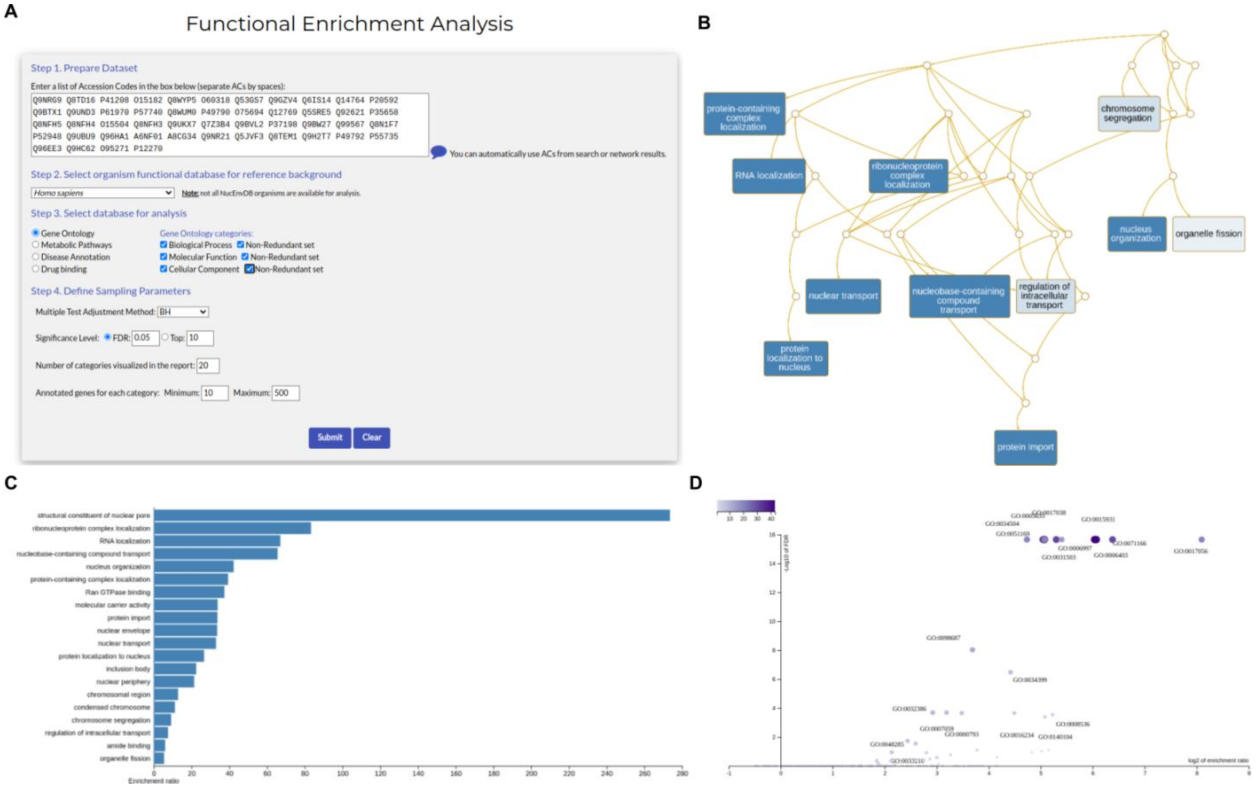

Figure S5. The functional enrichment interface of NucEnvDB. A) Input form. Users may paste a list of NucEnvDB accession codes, or automatically select them through the Protein Browser (see Figure 3). They can select the organism for analysis (in this case, *Homo sapiens*) and choose from a list of reference databases to perform enrichment. Finally, they can adjust the statistical test parameters to fine-tune their analysis. B-D) Enrichment results for GO terms, presented as a directed acyclic graph (B), a bar chart (C) or a volcano plot (D). All charts are interactive and can be adjusted based on the users' preferences.

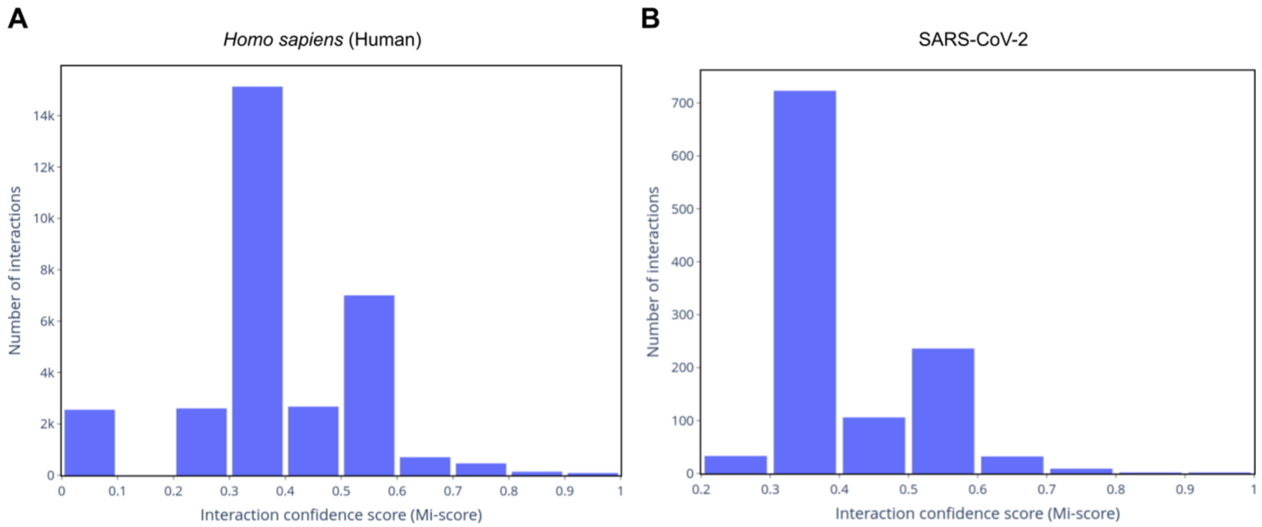

Figure S6. Distribution of protein-protein interaction scores (Mi-score) for the NE-localized proteins of *Homo sapiens* (A) and SARS-CoV-2 (B). The horizontal axis displays Mi-score values in intervals of 0.10. The vertical axis shows the number of interactions having a Mi-score value in each interval.

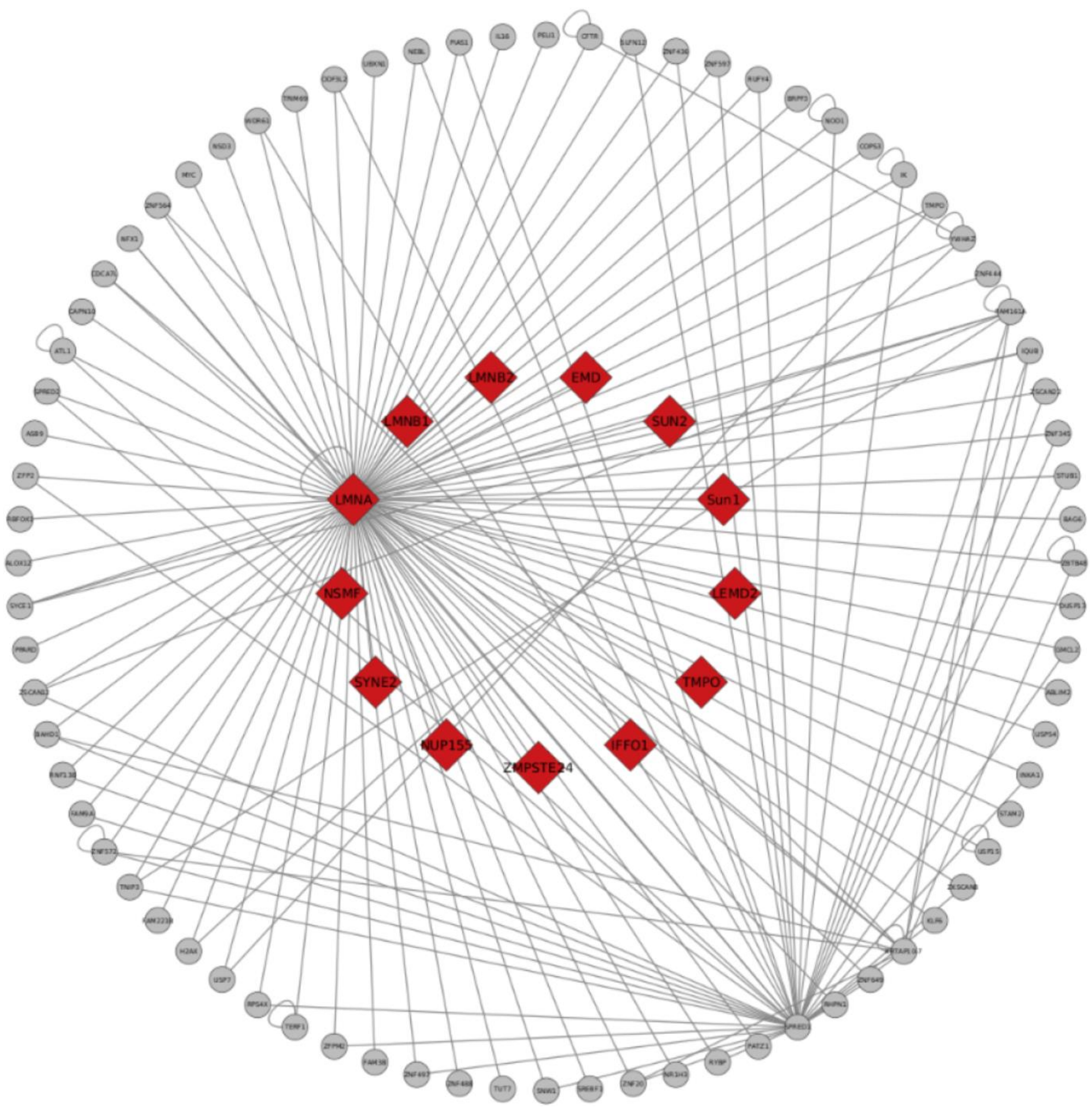

Figure S7. The human lamin A (LMNA) protein-protein interaction network. The network is shown using a grouped concentric layout, with LMNA and other NE proteins shown as diamonds (♦) and colored red, while proteins from other locations are shown as circles and colored grey.

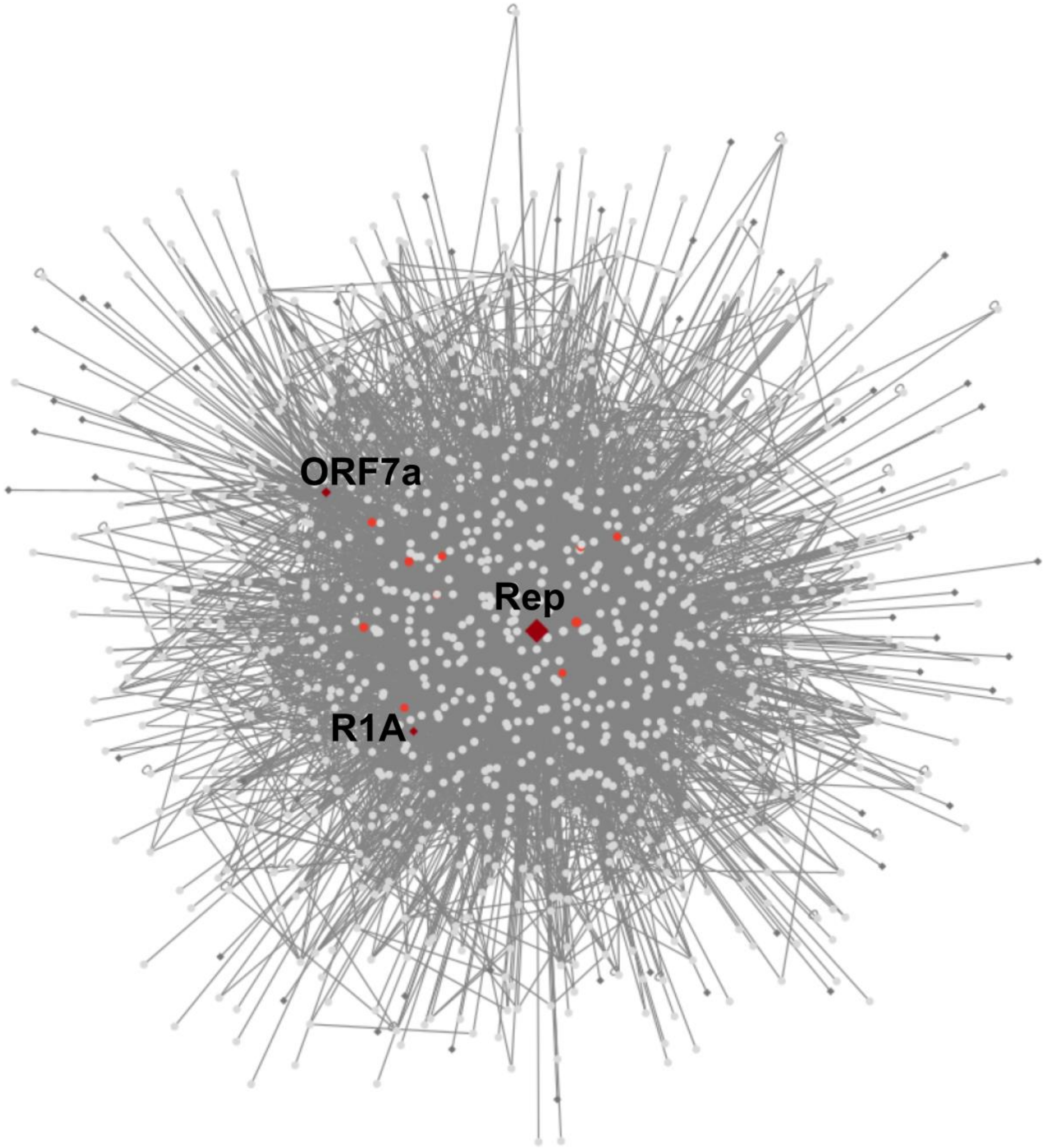

Figure S8. The SARS-CoV-2 - human interactome at the level of the nuclear envelope. Proteins localized at locations of the NE (either human or viral) are shown as diamonds (♦), while proteins from other locations are shown as circles.

Supplementary Tables

Table S1. Whole network functional enrichment results for GO (Biological process) and disease associations (DisGeNET &OMIM) for Case Study No.1.

Gene Set	Description	Size	Expect	Ratio	P-value	FDR
Gene Ontology – Biological Process						
GO:0006998	nuclear envelope organization	49	0.197012	30.45507	4.10E-08	6.38E-04
GO:0006997	nucleus organization	126	0.506601	13.81758	7.13E-07	0.003722
GO:0033044	regulation of chromosome organization	329	1.322792	7.55977	7.18E-07	0.003722
GO:0010256	endomembrane system organization	400	1.608257	6.21791	4.15E-06	0.01615
GO:0032434	regulation of proteasomal ubiquitin-dependent protein catabolic process	122	0.490518	12.23195	9.43E-06	0.025483
GO:0032435	negative regulation of proteasomal ubiquitin-dependent protein catabolic process	34	0.136702	29.26076	1.01E-05	0.025483
GO:0016570	histone modification	455	1.829393	5.466295	1.28E-05	0.025483
GO:0031062	positive regulation of histone methylation	38	0.152784	26.18068	1.59E-05	0.025483
GO:0031022	nuclear migration along microfilament	2	0.008041	248.7164	1.59E-05	0.025483
GO:0016569	covalent chromatin modification	468	1.881661	5.314453	1.64E-05	0.025483

GO:0070646	protein modification by small protein removal	288	1.157945	6.908789	1.95E-05	0.027517
GO:0031056	regulation of histone modification	142	0.570931	10.50914	2.24E-05	0.029046
GO:0031058	positive regulation of histone modification	87	0.349796	14.29405	2.59E-05	0.030926
GO:2000058	regulation of ubiquitin-dependent protein catabolic process	149	0.599076	10.01543	2.94E-05	0.032674
GO:2000059	negative regulation of ubiquitin-dependent protein catabolic process	47	0.18897	21.16735	3.74E-05	0.036527
GO:1905269	positive regulation of chromatin organization	94	0.37794	13.2296	3.76E-05	0.036527
GO:0021817	nucleokinesis involved in cell motility in cerebral cortex radial glia guided migration	3	0.012062	165.8109	4.77E-05	0.043518
GO:2001252	positive regulation of chromosome organization	164	0.659386	9.099381	5.04E-05	0.043518
GO:1901799	negative regulation of proteasomal protein catabolic process	53	0.213094	18.77105	6.03E-05	0.04696
GO:0051321	meiotic cell cycle	248	0.99712	7.020221	6.04E-05	0.04696
DisGeNET & OMIM(*)						
C0410189	Muscular Dystrophy, Emery-Dreifuss	4	0.019685	152.4038	4.39E-07	0.007728

C1860121	Decreased testosterone in males	28	0.137792	29.0293	9.42E-06	0.025946
C0270254	Hydrops of placenta	2	0.009842	203.2051	2.36E-05	0.025946
C0406585	Lethal tight skin contracture syndrome (disorder)	2	0.009842	203.2051	2.36E-05	0.025946
C0426433	Pinched nasal tip	2	0.009842	203.2051	2.36E-05	0.025946
C1136321	HIV-Associated Lipodystrophy Syndrome	2	0.009842	203.2051	2.36E-05	0.025946
C1835384	Loss of truncal adipose tissue	2	0.009842	203.2051	2.36E-05	0.025946
C1848760	Increased anterioposterior diameter of chest	2	0.009842	203.2051	2.36E-05	0.025946
C1848769	Overtubulated long bones	2	0.009842	203.2051	2.36E-05	0.025946
C4024993	Aplasia/Hypoplasia of the clavicles	2	0.009842	203.2051	2.36E-05	0.025946
C4025739	Acroosteolysis of distal phalanges (feet)	2	0.009842	203.2051	2.36E-05	0.025946
C1839653	Decreased cervical spine flexion due to contractures of posterior cervical muscles	2	0.009842	203.2051	2.36E-05	0.025946
C4280512	Inability to touch chin to chest	2	0.009842	203.2051	2.36E-05	0.025946
C0149744	Oral lesion	2	0.009842	203.2051	2.36E-05	0.025946
C4280289	Lesion of oral cavity	2	0.009842	203.2051	2.36E-05	0.025946
*275210	RESTRICTIVE DERMOPATHY, LETHAL	2	0.009842	203.2051	2.36E-05	0.025946
C1837757	Progressive acroosteolysis of the clavicle	3	0.014763	135.4701	7.06E-05	0.044887
C1848771	Prominent superficial vasculature	3	0.014763	135.4701	7.06E-05	0.044887

C1848773	Epidermal hyperkeratosis	3	0.014763	135.4701	7.06E-05	0.044887
C1856542	Prominent scalp veins	3	0.014763	135.4701	7.06E-05	0.044887
C1867114	Craniofacial disproportion	3	0.014763	135.4701	7.06E-05	0.044887
C3810018	Bilateral coxa valga	3	0.014763	135.4701	7.06E-05	0.044887
C4025078	Tapering pointed ends of distal finger phalanges	3	0.014763	135.4701	7.06E-05	0.044887
C4025270	Arteriosclerosis of small cerebral arteries	3	0.014763	135.4701	7.06E-05	0.044887
C4280505	Hardened artery wall in small cerebral arteries	3	0.014763	135.4701	7.06E-05	0.044887
C0085611	Atrial arrhythmia	3	0.014763	135.4701	7.06E-05	0.044887
C4025755	Primary atrial arrhythmia	3	0.014763	135.4701	7.06E-05	0.044887
C1846228	Absence of pubertal development	17	0.083659	35.85973	7.14E-05	0.044887

Table S2. Topological analysis results for the SARS-CoV-2 - human host-pathogen network of the NE.

**Parameter**	**Value**	**Parameter**	**Value**
Nodes	1052	*Avg. Clust. coeff.*	0.393
Edges	4868	*Density*	0.008
Avg. number of neighbors	8.928	*Heterogeneity*	3.458
Diameter	4	*Centralization*	0.888
Radius	2	*Connected components*	1
Avg. Path length	2.173	*GLay clusters*	11

Table S3. Whole network functional enrichment results for metabolic pathways (REACTOME), disease associations (DisGeNET) and drug binding (DrugBank).

Gene Set	Description	Size	Expect	Ratio	P-value	FDR
REACTOME metabolic pathways						
R-HSA-5663205	Infectious disease	382	25.928	2.8541	<2.2e-16	<2.2e-16
R-HSA-72766	Translation	291	19.751	3.5440	<2.2e-16	<2.2e-16
R-HSA-72312	rRNA processing	204	13.846	3.9722	<2.2e-16	<2.2e-16
hsa03013	RNA transport	166	11.267	3.5501	8.9606e-13	4.8965e-11
R-HSA-71291	Metabolism of amino acids and derivatives	370	25.114	2.3493	4.8383e-10	2.4837e-8
R-HSA-68886	M Phase	393	26.675	2.2868	7.3175e-10	3.6459e-8
R-HSA-72203	Processing of Capped Intron-Containing Pre-mRNA	243	16.493	2.6071	5.0234e-9	2.2394e-7
R-HSA-1852241	Organelle biogenesis and maintenance	294	19.955	2.4054	9.7815e-9	4.0414e-7
R-HSA-69242	S Phase	161	10.928	2.8368	9.9908e-8	0.0000033185
hsa04141	Protein processing in endoplasmic reticulum	165	11.199	2.5895	0.0000018576	0.000034204

DisGeNET diseases						
C0019693	HIV Infections	100	5.6151	3.9180	2.2596e-8	0.000051495
C1260899	Anemia, Diamond-Blackfan	16	0.89842	8.9045	8.0180e-7	0.00046900
C0700208	Acquired scoliosis	282	15.835	2.3366	9.7982e-7	0.00046900
C0855329	Electrocardiogram change	78	4.3798	3.4248	0.000022761	0.0039901
C1836296	Weakness of lower limb	27	1.5161	5.2767	0.000079862	0.0088077
C0270948	Neurogenic Muscular Atrophy	139	7.8050	2.5624	0.000088888	0.0088077
C0151889	Hyperreflexia	209	11.736	2.2155	0.00010347	0.0098255
C0376545	Hematologic Neoplasms	22	1.2353	5.6665	0.00013690	0.011143
C1138421	Spider Veins	17	0.95457	6.2855	0.00022052	0.014892
C0232744	Decreased liver function	24	1.3476	5.1943	0.00025188	0.014892
C4280655	Narrow head shape	76	4.2675	3.0463	0.00027444	0.014892
C4020855	Respiratory function loss	121	6.7943	2.5021	0.00039579	0.018238
C4020861	Depressed philtrum	21	1.1792	5.0883	0.00079777	0.033056
DrugBank Chemical Compounds						

DB11638	Artenimol	84	4.5312	6.1793	3.3307e-16	1.8685e-13
DB12695	Phenethyl Isothiocyanate	44	2.3735	4.2132	0.000079283	0.022239
DB00157	NADH	147	7.9297	2.3961	0.00024960	0.046675
